# The mental health of nursing students during the COVID-19 pandemic: Beneficial effects of mindfulness-based stress reduction

**DOI:** 10.1016/j.heliyon.2024.e32986

**Published:** 2024-06-13

**Authors:** Fariba Eslamimoghadam, Zahra Abedini, Ashraf Khoramirad

**Affiliations:** a Qom University of Medical Sciences, Qom, Iran; b Faculty Member of Qom University of Medical Sciences, Qom, Iran

**Keywords:** Mindfulness-based stress reduction, Stress, Anxiety, Depression, Nursing students

## Abstract

**Background:**

Following the COVID-19 global emergency, health students were faced with increased workloads, university closures, study interruptions, loss of peer support networks, and the challenges of volunteer work in hospitals. These factors caused health students to experience significant stress and anxiety, highlighting the necessity of psychological interventions for this group. Several studies have reported that a mindfulness-based stress reduction (MBSR) protocol offers valuable coping skills for traumatic events. This study aimed to investigate the impact of Mindfulness-Based Stress Reduction (MBSR) on stress, anxiety, and depression among nursing students at Qom University of Medical Sciences during the COVID-19 pandemic.

**Method:**

This experimental study was conducted on 72 nursing students from the nursing faculty of Qom University of Medical Sciences in 2020. The sampling was conducted using stratified sampling, and the allocation method employed was simple randomization. MBSR intervention based on social networks was implemented for the experimental group. The Stress-Anxiety-Depression Assessment Questionnaire (DASS-21) was completed by both groups before the study commenced, immediately after, and 2 months post-intervention. Data were analyzed in SPSS-16 using *t*-test, chi-square, and repeated measures analysis.

**Results:**

Analysis of variance with repeated measures showed that in the experimental group, the effect of time on the average score of stress, anxiety, and depression is significant *(p < 0.001).* The *t*-test showed significant differences between the two groups in terms of stress, anxiety, and depression scores immediately after the intervention *(p < 0.001)* and in the follow-up phase *(p < 0.001).*

**Conclusions:**

MBSR based on social networks is effective and applicable in reducing stress, anxiety, and depression among nursing students during the COVID-19 pandemic. It is suggested that MBSR should be included in the curriculum of nursing students so that they can have the necessary mental preparation to face acute critical conditions such as COVID-19.

## 1 Introduction

The COVID-19 pandemic has been one of the world's major health challenges in recent years, which has had significant consequences for the health system [1]. The nurses who were on the front lines fighting this pandemic were under intense physical and mental pressure [2]. The mental distress experienced by nurses is primarily characterized by sleep disorders, anxiety and depression symptoms, post-traumatic stress, difficulty in making decisions, and even physical symptoms [1].

During the COVID-19 pandemic, the heavier workload and life-threatening conditions have exacerbated mental stress among nurses, leading to an increase in the occurrence of mental illnesses among them [3]. One of the consequences of COVID-19 on the healthcare system was an escalation in violence against frontline workers, including nurses, leading to a rise in psychological issues like anxiety and depression among them [4]. Nursing students, like working nurses, were exposed to the aforementioned threats. They observed the critical conditions of the nurses and felt afraid and uncertain about their future careers. The increase in the number of hospitalized patients, the large number of sick nurses, and the extraordinary workload in the health system have led to an increasing demand for nursing staff on the front lines of medical centers. In these situations, nursing students were invited to meet the required needs. In addition, the changes in the educational system, such as closing classes and shifting to virtual education, have also imposed a lot of stress on students [[5], [6], [7]]. Studies conducted during the COVID-19 pandemic in different parts of the world indicated high levels of psychological distress among nursing students [5,8]. The findings of Thomas et al.'s study (2022) showed that nursing students experienced high levels of stress during the COVID-19 pandemic [7]. The findings of Hasanpour et al. (2021) in Iran also confirmed that nursing students experienced acute generalized anxiety disorder during the COVID-19 pandemic [6].

Stress is a crucial psychosocial element in the educational process that can impact students' academic performance and well-being [9,10]. Anxiety negatively impacts students' quality of life, education, and clinical performance, potentially leading them to withdraw from the nursing program [10]. Long-term anxiety and depression can also lead to physical and mental damage, reduced academic performance, a decrease in quality of life, interpersonal tensions, and other problems in students [11]. Studies show that mental health parameters are affected by emotion-oriented coping styles, including emotional responses and self-preoccupation. Therefore, an adaptive coping program such as mindfulness-based stress reduction (MBSR) can reduce psychological distress in nursing students [9,12].

Mindfulness-Based Stress Reduction (MBSR) is an eight-week evidence-based program that provides intensive mindfulness training to help individuals cope with stress, anxiety, depression, and pain [13,14]. MBSR was developed at the University of Massachusetts Medical Center in the 1970s by Professor Jon Kabat-Zinn. MBSR utilizes a combination of mindfulness meditation, body awareness, yoga, and exploration of patterns of behavior, thinking, feeling, and action [13]. Mindfulness can be understood as the non-judgmental acceptance and investigation of present experience, including body sensations, internal mental states, thoughts, emotions, impulses, and memories, to reduce suffering or distress and to increase well-being [15]. Mindfulness meditation is a method that cultivates attention skills, develops emotional regulation, and significantly reduces rumination and worry [13]. During the past decades, MBSR has been the subject of more controlled clinical research, which suggests its potential beneficial effects for mental health as well as physical health. The benefits of MBSR training for a variety of populations have been increasingly recognized in both academic literature and popular media. MBSR is one of the most frequently mentioned empirically supported interventions for reducing chronic pain, stress, anxiety, and depression. MBSR training encourages nonjudgmental observation and acceptance of all bodily sensations as they arise [16,17]. In this way, the study by Wylde et al. (2017) showed that MBSR reduces burnout and increases mindfulness and compassion in nurses [18]. Duarte et al. (2016) found that MBSR reduces stress, burnout, and fatigue, and increases life satisfaction in oncology nurses [19].

However in a study by Shabani, cognitive therapy based on mindfulness did not have a significant effect on perfectionism, and the intervention based on mindfulness did not show any difference compared to other interventions on the rumination [20]. In another study by Zuo et al., the mindfulness intervention did not have a significant impact on the mindfulness levels of the intervention group [21].

Considering the conflicting results regarding the effectiveness of MBSR, it is necessary to conduct more studies in this field. Furthermore, the high stress of the COVID-19 crisis, its globalization, uncertainties about treatment, travel restrictions, university closures, and uncertainty about the future of nursing employment have unprecedentedly affected nursing students psychologically. It is unclear whether MBSR is still effective in this group and under these circumstances. On the other hand, many colleges and universities were transitioning towards education based on social networks due to quarantine-related restrictions that have eliminated the possibility of delivering face-to-face interventions. This was while the implementation of MBSR through mobile and virtual networks had already been evaluated as feasible and effective. Therefore, this study aimed to assess the effect of mindfulness-based stress reduction through social networks on stress, anxiety, and depression among nursing students at the Faculty of Nursing, Qom University of Medical Sciences during the COVID-19 pandemic.

## 2 Material and methods

### 2.1 Study design and participants

This experimental study was conducted with a two-group design (experimental and control) at the nursing faculty of Qom University of Medical Sciences from February to April 2020. The sampling was conducted using stratified sampling across academic years and random sampling within each academic year. The allocation method employed was simple randomization. The sample size was estimated with a confidence level of 95 % and a test power of 95 %, considering 5 points for statistical significance. Initially, 32 participants were planned for each group. However, to account for a predicted 20 % sample attrition rate, 42 individuals were enrolled in the study. The study was conducted from February to April and did not interfere with the end-of-semester exams.

The inclusion criteria were: willingness and consent to participate in the study, being a Nursing student at least 5th semester, not taking psychiatric drugs, not having participated in a mindfulness program before. In the course of the study, the participants would have been excluded if they underwent a stressful life event(e.g. death of relatives, divorce – either for oneself or parents), took study leave, unwillingness to continue cooperation, severe infection with COVID- 19, not listening to all the files and not doing at least one of the exercises for at least 10 min a day during the intervention(for the experimental group).

### 2.2 Research design and steps

After obtaining the necessary permits, a list of nursing students in the 5th semester and above was prepared, including their phone numbers. First, the students were contacted by phone, and the objectives of the study were explained to them. Those who were eligible to participate in the study were then included in the sample list. After preparing a list of eligible students' names according to different academic years, 14 students from each year were selected through simple random sampling. In each set, 7 people were randomly selected for the experimental group and 7 for the control group. Finally, the study was conducted with 84 students. After sample attrition, statistical analysis was performed on 72 students who continued to participate until the end of the study (experimental group = 36, control group = 36).

### 2.3 Intervention

**Mindfulness-based stress reduction** (**MBSR**) is an eight-week evidence-based program that offers secular, intensive mindfulness training to assist people with stress, anxiety, depression, and pain [13,14] developed at the University of Massachusetts Medical Center in the 1970s by Professor Jon Kabat-Zinn, MBSR uses a combination of mindfulness meditation, body awareness, yoga, and exploration of patterns of behavior, thinking, feeling, and action [13]. Over the past decades, MBSR has been the subject of controlled clinical research and has been applied to both clinical and non-clinical populations.

In our study, MBSR was designed based on Kabat-Zinn's protocol (2003)(13), and the study of Borjalilu et al.(2019) [22] in Iran. MBSR in our study was implemented based on social networks due to the prevailing conditions during the COVID-19 pandemic, quarantine and closure of face-to-face classes, and the existing limitations for face-to-face interventions.

To implement the intervention, an initial orientation session was held in groups of 6–10 for the students in the experimental group. In this session, various materials related to stress were discussed, including the stress formation process, sources of stress, the physiology of stress (including the autonomic nervous system, hormones, and immune system), stress and diseases, and stress symptoms (both physical and mental symptoms). Basics and preliminaries about MBSR were also presented. Eight 10–20 min podcasts and two short videos were presented to the students over 8 days through a mobile phone messaging application. The eight podcasts include: The training videos included muscle relaxation techniques, such as tensing and relaxing muscle groups. Some of the podcasts include body scanning and deep breathing exercises accompanied by soothing sounds, such as nature sounds (waterfalls, flowing rivers, wind, and birds singing).

For each participant, the application available to them (Eita, WhatsApp, or Telegram) was used. The intervention was delivered by the interventionist, a Ph.D. in health psychology with experience in mindfulness. The participants in the experimental group were asked to listen to the audio files daily at their preferred time and to perform the exercises during the day or before bedtime whenever possible.

The research team ensured that the files and exercises were accessed every three days. Furthermore, students could contact the instructor if they had any questions or uncertainties. During the intervention, various instructional materials related to time management and life skills were provided to the control group. After collecting the data for the follow-up session, the MBSR instructional materials were sent to them. The research questionnaires were completed before the start of the intervention, at the end of the intervention (after the second week), and two months after the intervention. Afterward, the collected information underwent statistical analysis.

### 2.4 Data collection tool

The personal information questionnaire included age, gender, marital status, native status, academic term, student work experience, and overall grade point average.

To measure mental health parameters, the Depression, Anxiety, and Stress Scale (DASS-21) questionnaire by Lovibond & Lovibond used [23]. This questionnaire was created in 1995 to measure depression, anxiety, and stress and consists of 21 items. The DASS-21 questionnaire consists of three components, and each of its subscales contains seven questions. The final score of each subscale is obtained by summing the scores of its component questions. To score the questionnaire, each question is graded from 0 (does not apply to me at all) to 3 (completely applies to me). Since the DASS-21 is a shortened version of the original scale with 42 items, the final score for each subscale should be doubled [23]. The total score obtained from each component ranges from 0 to 42. Higher scores indicate higher levels of depression, anxiety, and stress. In Iran, Sahebi and colleagues have validated this tool, and Cronbach's alpha coefficient showed good reliability. In a study conducted in Iran in 2017 by Aazami et al. to investigate the confirmatory factor structure of the Depression, Anxiety, and Stress Scale (DASS) in students, the results indicated that the 3-factor model (depression-anxiety-stress) for Iranian students was confirmed through confirmatory factor analysis. The fit indices suggested a good fit of the data with the 3-factor model. The negative correlation of this scale with the short form of Riff's psychological well-being scale also confirmed the validity of this scale [24]. In another study in Iran in 2008, the reliability of DASS scales was confirmed by examining internal consistency coefficients and retest coefficients [25]. In our study, Cronbach's alpha was estimated at 0.92 for depression, 0.94 for anxiety, and 0.86 for stress.

### 2.5 Statistical analysis

After collecting the data, it was entered into SPSS (Version 16). To compare quantitative variables in two groups, the independent *t*-test was used, and for comparing qualitative variables, the Chi-square test was employed. Considering the data distribution, the independent *t*-test was used to compare the average scores of mental health parameters in the two groups. Also, the one-way analysis of variance test with repeated measures was used to assess the average scores of mental health parameters at three time points. The p-value, which was less than 0.05 (p < 0.05), was considered statistically significant.

### 2.6 Ethical considerations

The code of ethics was obtained from Qom University of Medical Sciences (IR.MUQ.REC.1401.014), and all the ethical principles of the intervention plans were complied with. The intervention for the control group was implemented after data collection. The confidentiality of information was also maintained. Participation in the study was voluntary, and participants could withdraw at any time if they no longer wished to cooperate.

## 3 Results

In this study, 72 subjects, divided into an experimental group (36 people) and a control group (36 people), reached the final analysis stage. No statistically significant difference was observed between the demographic variables in the two groups *(p < 0.05)* (Table 1). Variance analysis with repeated measures revealed that in the experimental group, the impact of time on the average scores of stress, anxiety, and depression is significant. There is a statistically significant difference between the scores of stress, anxiety, and depression at three-time points *(p < 0.001).* The *t*-test indicated that before the intervention, there was no significant difference between the two groups in terms of stress, anxiety, and depression *(p = 0.231, p = 0.08*, and *p = 0.67*, respectively). However, immediately after the intervention *(p < 0.001)* and during the follow-up phase *(p < 0.001)*, a significant difference between the two groups was observed (refer to Table 2, Table 3, Table 4).

**Table 1:** Characteristics of participants in two groups.

group variable		experimental (n = 36)	control (n = 36)	*p-value*
		frequency (percent)	frequency (percent)	
**gender**	**male**	**17(47.2)**	**19(52.80)**	***p = 0.63***
	**female**	**19(52.8)**	**17(47.2)**	
**marital status**	**single**	**26(72.2)**	**26(72.2)**	***p = 1.00***
	**married**	**10(27.8)**	**10(27.8)**	
**native**	**yes**	**29(80.6)**	**27(75)**	***p = 0.57***
	**no**	**7(19.4)**	**9(25)**	
**academic year**	**3**	**23(63.9)**	**23(63.9)**	***p = 1.00***
	**4**	**13(36.1)**	**13(36.1)**	
**student work**	**yes**	**12(33.3)**	**13(36.1)**	***p = 0.80***
	**no**	**24(66.77)**	**23(63.9)**	
**age**	**mean** ± **standard deviation**	**2.01** ± **21.49**	**2.13 ± 22.99**	***t = 0.511*** ***p = 0.61***
**overall GPA**	**mean** ± **standard deviation**	**1.41** ± **17.06**	**17.1 ± 16.65**	***t = 1.356*** ***p = 0.17***

*Chi Square Test ** T-test.

**Table 2:** The results of repeated measures analysis for total scores of Stress.^a^.

Variable	pretest		Posttest		Follow-up		*p-value*^b^		
	Intervention	Control	Intervention	Control	Intervention	Control	Group	Time	Time-Group
Stress	29.20 ± 5.28	26.22 ± 10.20	15.20 ± 2.93	24.10 ± 9.70	7.02 ± 2.24	23.32 ± 9.60	*<0.001*	*<0.001*	*<0.001*
Effect size(Cohen's d)	0.287		−1.083		−2.180				
*p-value*^c^	*0.231*		*<0.001*		*<0.001*				

a Data presented as mean±SD, SD: Standard deviation.

b Repeated measures analysis.

c t-test.

**Table 3:** The results of repeated measures analysis for total s^c^ores of Anxietys^a^.

Variable	pretest		Posttest		Follow-up		*p-value*^b^		
	Intervention	Control	Intervention	Control	Intervention	Control	Group	Time	Time-Group
Anxiety	28.32 ± 5.75	26.94 ± 7.44	13.20 ± 3.08	24.76 ± 4.84	6.16 ± 1.82	22.38 ± 4.59	*<0.001*	*<0.001*	*<0.001*
Effect size(Cohen's d)	0.104		−1.422		−2.322				
*p-value*^c^	*0.08*		*<0.001*		*<0.001*				

a Data presented as mean ± SD, SD: Standard deviation.

b Repeated measures analysis.

c t-test.

**Table 4:** The results of repeated measures analysis for total scores of Depression.^a^.

Variable	pretest		Posttest		Follow-up		*p-value*^b^		
	Intervention	Control	Intervention	Control	Intervention	Control	Group	Time	Time-Group
**Depression**	28 ± 5.60	26.94 ± 4.81	14.38 ± 3.56	24.54 ± 5.32	6.54 ± 1.95	23.94 ± 5.99	*<0.001*	*<0.001*	*<0.001*
Effect size(Cohen's d)	0101		−1.123		−1.951				
*p-value*^c^	*0.670*		*<0.001*		*<0.001*				

a Data presented as mean ± SD, SD: Standard deviation.

b Repeated measures analysis.

c t-test.

## 4 Discussion

This study was conducted to reveal the impact of MBSR via social networks on the mental health of nursing students during the COVID-19 pandemic. The initial findings showed that the levels of stress, anxiety, and depression in both groups were at a severe level at the beginning of the study and there was no significant difference in scores between the two groups in terms of depression, anxiety, and stress before the intervention. Since the subjects were selected randomly, the lack of difference in the average scores of the groups was expected and is logical.

Consistent with our results, in previous studies, 70 % of nursing students suffered from moderate to severe anxiety when returning to university during the time of COVID-19 [26]. In a systematic review in 2021, the findings indicated the prevalence of stress, anxiety, and depression among health professionals during the COVID-19 pandemic in the range of 44.86 %, 41.42 %, and 37.12 % respectively [27]. In another study on nurses in China, many sources of stress were identified for nurses on the front line of COVID-19 [28]. In studies conducted in Iran on nurses and healthcare workers during the Covid-19 pandemic, high levels of anxiety and depression were reported and the need to design and implement stress management interventions for these vulnerable groups was emphasized [29,30].

Other findings showed that in the post-test and follow-up phases, stress, anxiety, and depression in the experimental group were significantly lower than in the control group, which shows the positive effects of MBSR in reducing stress, anxiety, and depression. Consistent with our findings Bennett et al. (2016) found that the implementation of MBRS reduced depression in students [31]. Also, the results of Song et al. [16] in a clinical trial study in Korea are consistent with our findings, they found the implementation of MBRS during 8 sessions reduced depression, anxiety, and stress in nursing students [9]. Dobie et al. (2016) also found in a one-group pretest–posttest study that the implementation of MBRS during 8 weeks and in 15-min sessions improved the mental health of professionals who provide health care services (nurses, social workers, and occupational therapists) [32]. The findings of a systematic reviw during the COVID-19 pandemic indicated the effectiveness of mindfulness-based interventions on mental health [33]. Belen et al. reported that the fear of COVID-19 has an inverse relationship with mindfulness. Additionally, they found a direct and significant relationship between the fear of COVID-19 and anxiety and depression [34]. Other studies in the same field confirmed that MBSR is effective in controlling stress, anxiety, and depression [[35], [36], [37]].

In explaining the findings, MBSR helps students overcome the stress caused by Covid-19 in some ways. First, by developing mindful attention. Mindful attention is characterized by the intentional directing of awareness to activities focused on the present for sustained periods of time. In contrast, a complete lack of mindful attention is characterized by a state of conflicted awareness caused by immediate reactions to past memories, worries about the future, or negative thoughts or feelings in the present. Students in the MBSR group may have been better able to focus their attention on the ongoing performance task, whereas the control group may have been more easily distracted by the stressors of COVID-19. Another explanation involves developing acceptance and regulation of feelings and thoughts. Mindfulness encourages people to perceive events and occurrences in a non-judgmental manner and view them as opportunities for growth.

[38]. When students practice mindfulness in their daily lives, they recognize the time spent in a normal state. This practice provides them with an opportunity to enter and live in the present, thereby breaking the chain of events that lead to unpleasant and anxiety-inducing feelings. MBSR also encourages individuals to respond to their current situation creatively and helps them break free from abnormal reactions. Moreover, physical exercises in MBSR enhance the body's sensitivity to receiving messages and reduce mental confusion. In this way, despite the intense emotions associated with their jobs, nursing students in the MBSR group can maintain their normal lives, enjoy good mental health, and take corrective actions when faced with stressful situations [39,40]. It can be concluded that mindfulness helps individuals to absorb the present moment as it is, instead of being involved in the past or future. Mindfulness is voluntary and when persons cultivate it, they become more aware of the present reality and the available options. Moreover, mindfulness helps people to observe situations non-judgmentally, and instead of interpreting events as terrible, which is anxiety-ridden, experience them with a non-judgmental observation and respond to them by focusing. so they can focus on solving the problem instead of getting involved in stress and anxiety [17,41].

Some previous studies do not confirm our results and did not report significant effects of MBSR on increasing mindfulness [20,21]. Although the disparity in findings may be attributed to factors such as sample size, tools used, duration of the intervention, or the characteristics of the population in which the intervention was conducted, the significant aspect is that the effectiveness of MBSR in our study related to a comprehensive and holistic approach. This approach addressed the stressors faced by nursing students during the Covid-19 crisis and their specific coping mechanisms, and facilitated access to training materials through available applications for students. In our intervention, we adjusted the time and place of the exercises based on the students' personal preferences and needs that making it easier for them to participate in the intervention. Previous studies may have lacked this appropriate approach, leading to less significant results.

The findings of our study showed that mindfulness can be implemented virtually and through mobile applications, and it can be effective in reducing stress, anxiety, and depression among nursing students in critical situations such as Covid-19. Previously, Cox et al. reported positive results regarding the applicability and effectiveness of telephone-based mindfulness interventions on distress symptoms [42]. Review studies have also concluded that MBSR can be effective in virtual treatment protocols [43]. In another study, Online MBSR had good results on the well-being of nurses working in COVID-19 care units [44]. Our findings provided further confirmation of the effectiveness of mobile-based mindfulness interventions.

## 5 Limitations and suggestions for further studies

There was a possibility of disclosing information from the experimental group to the control group, which was mitigated by informing the experimental group subjects that the data and instructional files were for their personal use only and should not be shared. Since the sample was predominantly selected from a specific nursing school, the findings may not be readily generalizable to other areas or diverse educational settings. The duration of the intervention may affect the results of the study. The absence of long-term follow-up assessments hinders the ability to determine the enduring effects of the MBSR intervention. Ensuring stability and quality control of online intervention programs may be challenging. Participants may have engaged and participated in the intervention in different ways, which could have potentially impacted the stability of the study results. In addition to the limitations of self-reporting, the absence of other objective measures (such as physiological indices) to evaluate levels of anxiety, stress, and depression may limit the interpretation of results.

It is suggested that MBSR be included in the curriculum of nursing students so that they are adequately prepared to handle acute critical situations like COVID-19. It is suggested that future studies examine the details of MBSR components to determine which components have a greater impact on mental health. It is also suggested that other studies investigate the effect of homework completion and address the question of whether better outcomes will be achieved by extending the duration of homework assignments. Further studies involving students in health-related fields and utilizing more accurate methods to assess psychological parameters are suggested.

## 6 Conclusion

In conclusion, we found that the MBSR is applicable and beneficial in situations like the COVID-19 pandemic, especially when the stress levels of healthcare students are high. In situations where face-to-face psychological interventions are not possible, such as during quarantine, MBSR can be implemented through social networks and prove effective in reducing stress, anxiety, and depression.

## Ethical approval statement

The study was conducted according to the guidelines of the Declaration of Helsinki, and approved by the Institutional Ethics Committee of Qom University of Medical Sciences with the approval number **IR.MUQ.REC.1401.014.**

## Data availability statement

Data will be made available on request.

## CRediT authorship contribution statement

**Fariba Eslamimoghadam:** Writing – review & editing, Visualization, Methodology, Data curation. **Zahra Abedini:** Writing – review & editing, Project administration, Methodology, Investigation, Formal analysis. **Ashraf Khoramirad:** Writing – review & editing, Project administration, Methodology, Investigation, Funding acquisition, Conceptualization.

## Declaration of competing interest

The authors declare the following financial interests/personal relationships which may be considered as potential competing interests:Ashraf Khoramirad reports financial support was provided by Qom University of Medical Sciences. If there are other authors, they declare that they have no known competing financial interests or personal relationships that could have appeared to influence the work reported in this paper.
